# Cerium and samarium blocked antioxidant enzymes in wheat plants

**DOI:** 10.1038/s41598-023-35561-2

**Published:** 2023-05-22

**Authors:** Amirhossein Kazemzadeh Haghighi, Maryam Rezayian, Vahid Niknam, Mohammad Reza Ganjali, Masoud Mirmasoumi

**Affiliations:** 1 Department of Plant Biology, School of Biology, College of Science, University of Tehran, Tehran, 14155 Iran; 2 Center of Excellence in Medicinal Plant Metabolites, Tarbiat Modares University, Tehran, Iran; 3 Center of Excellence in Electrochemistry, School of Chemistry, University of Tehran, Tehran, Iran

**Keywords:** Physiology, Plant sciences

## Abstract

This work was conducted to study positive and negative impacts of cerium (Ce) and samarium (Sm) on two cultivars (Arta and Baharan) in wheat plant. Symbols of stress such as proline, malondialdehyde (MDA) and antioxidant enzymes, which may be complicated in the suppression responses of plants, were also studied. Wheat plants were exposed to 0, 2500, 5000, 7500, 10,000 and 15,000 μM of Ce and Sm for 7 days. The growth enhanced in plants treated with lesser Ce and Sm concentration (2500 μM) and declined in plants treated with upper concentrations as compared to untreated plants. The treatment with 2500 μM of Ce and Sm increased dry weigh in Arta by 68.42 and 20% and in Baharan by 32.14% and 27.3%. Thus, Ce and Sm had hormesis effect on growth in wheat plants. According to plant’s growth parameter patterns, Arta cultivar had more sensitive to Sm than to Ce, whereas Baharan cultivar had sensitive to Ce than to Sm. Our results indicated impact of Ce and Sm on proline accumulation depended on the dosage of Ce and Sm. It was observed that Ce and Sm accumulated in wheat plants at higher exposure doses. Increment of MDA content by Ce and Sm treatments showed that these metals caused oxidative stress in wheat plants. Ce and Sm blocked enzymatic antioxidant system (superoxide dismutases, peroxidase and polyphenol peroxidase) in wheat. In wheat plants treated with lower Ce and Sm concentrations higher amounts of non-enzymatic antioxidant metabolites were detected. Thus, we showed the potential negative impact of unsuitable utilization of REEs in plants and suggested growth and interruption in physiological and biochemical mechanisms as a possible factor to recognize the underlying toxicological processes.

## Introduction

Rare earth elements (REEs), including yttrium, strontium and 15 lanthanides, have become an important commodity in terms of their enhanced application in industrial uses, such as electrodes and magnets^1^. In current years, there has been an enhancing notice in the study of the REEs and their uses in medicine, agriculture and biotechnology^2^. It has been described that the application of suitable concentration of REEs can stimulate seed germination and root growth, enhance chlorophyll content, increase tolerance, and consequently enhance production of crops^3^. REEs have been broadly used such as fertilizers to expand the quality and yield of plants. Unfortunately, negative impacts on crops have been witnessed due to high concentration of REEs. So far, the toxicity impacts of REEs was chiefly estimated according to phenotypic alternations, but information gaps still are regarding their metabolic impact^4^.

Cerium (Ce), the most abundant member of the trivalent metallic element cluster, is considered REEs that have and like ionic radii to magnesium and calcium and stable oxidation state (+ 3)^5^. Samarium (Sm) is an effulgent lustrous element with an atomic number of 62, molecular weight of 150.36 and oxidation level of + 3/ + 2^6^. Effect of REEs on physiological processes has been described in various plants. Lanthanum (La) increased maize yield by stimulating antioxidants enzymes, endogenous hormone and photosynthetic features at reproductive stages^7^. A high level of La was caused an increment in oxidative stress, malondialdehyde (MDA) level and hydrogen peroxide (H_2_O_2_) content in the seeds of rice^8^. Ce enhanced plant height, seed germination, root growth and biomass^9^.

REEs leaded the extreme generation of reactive oxygen species (ROS) and oxidative damage in plants, and cause severe injury to biological processes, causing programmed cell death. The negative impacts of ROS in plants are suppressed by an integrated antioxidant system. Its components are divided into enzymatic ones include catalase (CAT), superoxide dismutases (SOD), ascorbate peroxidase (APX) and peroxidase (POX) and non-enzymatic ones, such as phenolic compounds, ascorbate, glutathione and tocopherol^10^. The enhancement of flavonoid production and phenolic compounds is one of defense strategies in plants against the stress caused by REEs^11^.

Wheat (*Triticum aestivum* L.) is one of the chief sources of protein and calories and one of the main cereals in the world. About 82% and 85% of the worldwide population relates with wheat for protein and basic calories, respectively^12^. Also, this plant is applied in the production of different types of wheat products, such as steamed breads, flat, leavened bread, cakes, biscuits, pasta and noodles. Beyond its application for human utilization, wheat is also used for the increase of non-edible products such as fuel. Production of wheat is severely influenced by adverse environmental conditions^13^. Wheat is an experiment crop because it is one of the key economic plants and is cultivated on a universal scale. Moreover, wheat is commonly used such as an ecotoxicological pointer^14^.

Barely any data is available about the impact of Sm on growth and antioxidant response in wheat plant. In this regard, the purpose of this present work was to study the effects of Sm and Ce on growth in wheat plant. Aiming to better comprehend the behavior of this plant in the presence of two REEs, tolerance ability to these elements was evaluated. Also, antioxidant systems include enzymatic and non-enzymatic, proline accumulation, MDA content such as oxidative stress indicator and hydrogen peroxide (H_2_O_2_) content of wheat plant were assessed to determine the biological functions and physiological processes of wheat to REEs exposure. This work will involve to a deeper empathetic of the negative impacts caused by extreme treatment of REEs.

## Results

### Biomass and accumulation of proline

After 21 days of Ce or Sm exposure, the growth parameters of the wheat plants showed significant alternations when treated with various concentrations of REE (Tables 1, 2). Plant FW increased significantly by 56.43% in Arta and 25.4% in Baharan at 2500 µM Ce treatment, respectively, as compared to control. Other levels of Ce treatment declined plant FW in two cultivars and the rate of reduction was the highest levels in 15,000 µM Ce-treated plants. Ce treatment at 2500 µM enhanced (68.42%) plant DW in Arta cultivar, but 15,000 µM Ce decreased (47.37%) this parameter as compared to control. Plant DW enhanced with 2500 µM Ce by 32.14% in Baharan cultivar, but plant DW of Baharan cultivar reduced by 7.14, 7.14, 39.28 and 67.85% with the application of 5000, 7500, 1000 and 15,000 μM Ce, respectively, in comparison with the control. However, only with 15,000 μM treatment there was significant difference with respect to the control. In Arta cultivar, the height of the plants treated with 2500 to 10,000 µM Ce were not significantly affected. However, the plant height was significantly reduced in Arta cultivar for applied 15,000 µM Ce concentration when compared to the control. Plant height declined in Baharan cultivar with increasing Ce concentration. Root length diminished in Arta cultivar at 7500, 10,000 and 15,000 µM Ce, but different concentrations of Ce did not induce any significant changes in root length. The root length in Baharan cultivar decreased significantly with the application of all levels of Ce, in comparison with the control. Contrary to the effect on root length, Ce treatment had no influence on shoot length in two cultivars (Table 1).

**Table 1:** Impact of Ce on growth parameters in wheat plants.

Ce (µM)	Cultivar	Plant FW (g plant^–1^)	Plant DW (g plant^–1^)	Plant height (cm)	Root length (cm)	Shoot length (cm)
0	Arta	2.02 ± 0.460^bc^	0.196 ± 0.040^c-f^	48 ± 9.165^b-d^	19.6 ± 6.70^bc^	28.4±2.468^b-d^
2500		3.16 ± 0.238^a^	0.326 ± 0.039^ab^	50.5 ± 5.392^bc^	22 ± 2.516^b^	28.5±3.5^b-d^
5000		1.43 ± 0.194^c-e^	0.206 ± 0.013^c-e^	44.5 ± 2.753^b-e^	12.5 ± 1.755^b-d^	32±1.040^a-c^
7500		1.25 ± 0.151^c-f^	0.210 ± 0.208^cd^	41.833 ± 1.691^b-e^	9.433 ± 0.788^d^	32.4±1.248^a-c^
10,000		1.09 ± 0.226^d-f^	0.213 ± 0.033^cd^	35.666 ± 2.682^de^	8.266 ± 1.134^d^	27.4±1.646^c-e^
15,000		0.55 ± 0.066^f^	0.106 ± 0.017^ef^	32.266 ± 3.266^e^	9.033 ± 1.583^d^	23.23±2.411^de^
0	Baharan	2.56 ± 0.185^ab^	0.286 ± 0.043^a-c^	74 ± 6^a^	41.9 ± 5.901^a^	32.1±0.152^a-c^
2500		3.21 ± 0.375^a^	0.370 ± 0.058^a^	56 ± 2.309^b^	19.766 ± 0.788^bc^	36.233±1.920^a^
5000		1.73 ± 0.321^cd^	0.266 ± 0.029^b-d^	47.166 ± 3.678^b-d^	14 ± 2.516^b-d^	33.166±1.301^a-c^
7500		1.34 ± 0.148^c-e^	0.266 ± 0.020^b-d^	44.733 ± 2.395^b-e^	10.5 ± 0.763^ cd^	34.233±1.794^ab^
10,000		0.856 ± 0.103^ef^	0.176 ± 0.017^d-f^	41.666 ± 0.666^c-e^	10.5 ± 0.763^ cd^	31.166±0.833^a-c^
15,000		0.496 ± 0.029^f^	0.096 ± 0.008^f^	30.666 ± 4.226^e^	8.833 ± 1.922^d^	21.833±2.315^e^

Values are means ± SE of three replicates. Different letters indicated significant (p < 0.05) differences.

**Table 2:** Impact of Sm on growth parameters in wheat plants.

Sm (µM)	Cultivar	Plant FW (g plant^–1^)	Plant DW (g plant^–1^)	Plant height (cm)	Root length (cm)	Shoot length (cm)
0	Arta	2.506 ± 0.199^ab^	0.303 ± 0.032^cd^	53.8 ± 0.435^a-c^	22.333 ± 1.013^bc^	31.466 ± 1.278^a-c^
2500		3.266 ± 0.135^a^	0.360 ± 0.01^bc^	57.6 ± 3.780^ab^	26.666 ± 3.527^ab^	30.933 ± 1.484^a-c^
5000		2.666 ± 0.377^ab^	0.333 ± 0.029^b-d^	53.566 ± 4.330^a-c^	19.9 ± 3.044^b-d^	33.666 ± 1.847^ab^
7500		2.18 ± 0.740^a-c^	0.29 ± 0.011^c-e^	54.6 ± 1.908^a^	18.766 ± 1.398^c-e^	35.833 ± 0.935^a^
10,000		0.83 ± 0.061^d^	0.166 ± 0.024^fg^	38.566 ± 1.433^de^	11.4 ± 1.242^ef^	27.166 ± 0.666^c^
15,000		0.723 ± 0.127^d^	0.126 ± 0.0371^g^	35.4 ± 1.594^e^	9.233 ± 0.338f.	26.166 ± 1.540^c^
0	Baharan	2.856 ± 0.112^ab^	0.33 ± 0.01^b-d^	56.5 ± 1.322^a-c^	21.933 ± 1.212^bc^	34.566 ± 1.026^ab^
2500		3.396 ± 0.362^a^	0.42 ± 0.02^ab^	64.166 ± 5.783^a^	29.833 ± 4.693^a^	34.333 ± 1.092^ab^
5000		3.47 ± 0.625^a^	0.47 ± 0.052^a^	55.5 ± 2.886^a-c^	18.666 ± 1.369^c-e^	36.833 ± 1.540^a^
7500		1.913 ± 0.624^b-d^	0.256 ± 0.039^c-f^	48.166 ± 5.333^b-d^	17.333 ± 1.589^c-e^	30.833 ± 3.982^a-c^
10,000		1.156 ± 0.386^cd^	0.236 ± 0.063^d-f^	45.666 ± 4.977^c-e^	16.333 ± 2.587^c-f^	29.333 ± 2.803^bc^
15,000		0.856 ± 0.076^d^	0.183 ± 0.028^e-g^	42.5 ± 1.755^de^	13.666 ± 1.424^d-f^	28.833 ± 1.641^bc^

Values are means ± SE of three replicates. Different letters indicated significant (p < 0.05) differences.

In Arta cultivar, plant FW and plant DW were augmented by 17.8 and 20% for 2500 µM Sm, respectively. The reduction in Arta cultivar treated with 7500, 10,000 and 15,000 µm Sm was 12.8, 66.8 and 71.2% in plant FW and 3.3, 46.6 and 60% in plant DW compared to untreated plants, respectively. Plant FW in Baharan cultivar enhanced by 28 and 21.4% with application of 2500 and 5000 µM Sm. Other concentrations of Sm (7500, 10,000 and 15,000 µm Sm) caused 32.14, 58.93 and 69.64% decreases in plant FW of Baharan cultivar in compared with control groups, respectively. In Baharan cultivar, there was an increment of 27.3 and 42.42% in the plant DW with the addition of 2500 and 5000 μM Sm. Plant DW in Baharan cultivar declined in plants treated with high levels of Sm, in comparison with the control. These reductions were 24.24% for 7500 μM, 30.30% for 10,000 μM and 45.45% for 15,000 μM. In Arta cultivar, the plant height and root length were not significantly affected by the application of 2500–7500 µM Sm, compared with the control. On the other hand, the application of 10,000 and 15,000 μM Sm reduced the plant height and root length in Arta cultivar in comparison with the control. Conversely, the shoot length in Arta cultivar showed a significant trend with the application of Sm. The plant height and root length in Baharan cultivar did not change significantly at Sm concentrations up to 10,000 µM, while these parameters dropped with highest level of Sm (15,000 µM). Also, no significant difference was found in the shoot length between the Sm-treated and control plants in Baharan cultivar (Table 2).

No significant impacts on proline content were detected in all treatment of Ce in Arta cultivar, except for plants treated with 15,000 µM Ce. The 15,000 µM Ce treatment enhanced proline accumulation by 844 fold in Arta cultivar. In Baharan cultivar, low and moderate concentrations of Ce (2500–7500 µM) had no significant impact on proline content as compared to that of control, whereas high Ce concentrations (10,000 and 15,000 µM) caused a significant rise by 54.2 and 101.2 fold in proline content, respectively (Table 3). Proline content was not significantly affected by 2500–7500 µM Sm treatment in Arta cultivar relative to that of the control treatment. The exposure of 10,000 and 15,000 µM Sm caused significant elevation by 16 and 33.12 fold of proline content in Arta cultivar, displaying an increasing trend with exposure dose. Treatments with 7500 µM Sm in Baharan cultivar induced a significant enhancement in proline content, whereas other treatments did not cause any significant impact on proline content (Table 4).

**Table 3:** Impact of Ce on proline content, H_2_O_2_ content and MDA content in wheat plants.

Ce (µM)	Cultivar	Proline content (µg g^–1^FW)	H_2_O_2_ content (µg g^–1^FW)	MDA content (nmol g^–1^FW)
0	Arta	0.817 ± 0^d^	8.552 ± 0.034^e^	0.0077 ± 0^c^
2500		1.295 ± 0.670^d^	9.043 ± 0.045^d^	0.0092 ± 0^c^
5000		0.817 ± 0^d^	10.208 ± 0.055^a^	0.0151 ± 0.0001^b^
7500		32.915 ± 3.064^d^	7.408 ± 0.019^f^	0.0256 ± 0.0008^a^
10,000		4.061 ± 1.810^d^	7.457 ± 0.034^f^	0.0251 ± 0.0003^a^
15,000		689.530 ± 0^a^	5.911 ± 0.047^h^	0.0275 ± 0.0002^a^
0	Baharan	3.036 ± 0.171^d^	9.946 ± 0.077^b^	0.0085 ± 0^c^
2500		5.097 ± 1.911^d^	8.400 ± 0.032^e^	0.0072 ± 0.0004^c^
5000		3.469 ± 2.031^d^	9.513 ± 0.036^c^	0.0149 ± 0.0002^b^
7500		2.883 ± 0.305^d^	5.304 ± 0.072^i^	0.0189 ± 0.0004^b^
10,000		163.823 ± 2.801^c^	5.204 ± 0.142^i^	0.0249 ± 0.0046^a^
15,000		308.327 ± 131.283^b^	6.794 ± 0.072^g^	0.0240 ± 0.0002^a^

Values are means ± SE of three replicates. Different letters indicated significant (p < 0.05) differences.

**Table 4:** Impact of Sm on proline content, H_2_O_2_ content and MDA content in wheat plants.

Ce (µM)	Cultivar	Proline content (µg g^−1^FW)	H_2_O_2_ content (µg g^−1^FW)	MDA content (nmol g^−1^FW)
0	Arta	12.643 ± 1.030^d^	11.454 ± 0.051^c^	0.0397 ± 0.0006^g^
2500		20.258 ± 0.381^d^	10.504 ± 0.046^d^	0.0412 ± 0.0007^g^
5000		11.960 ± 0.591^d^	10.095 ± 0.035^e^	0.0536 ± 0.0009^f^
7500		18.596 ± 2.037^d^	8.820 ± 0.031^f^	0.0880 ± 0.0027^d^
10,000		202.243 ± 1.431^bc^	7.882 ± 0.073^i^	0.1286 ± 0.0011^b^
15,000		418.696 ± 93.619^a^	6.496 ± 0.110^k^	0.1443 ± 0.0005^a^
0	Baharan	122.006 ± 3.527^c^	13.540 ± 0.035^a^	0.0445 ± 0.002^g^
2500		32.505 ± 0.870^d^	12.137 ± 0.054^b^	0.0428 ± 0.0026^g^
5000		21.373 ± 0.290^d^	8.182 ± 0.023^h^	0.0778 ± 0.0022^e^
7500		223.926 ± 6.280^b^	8.442 ± 0.015^g^	0.0847 ± 0.0011^d^
10,000		23.696 ± 1.453^d^	8.005 ± 0.0788^i^	0.0999 ± 0.0045^c^
15,000		162.813 ± 2.578^bc^	6.845 ± 0.077^j^	0.1266 ± 0.0010^b^

Values are means ± SE of three replicates. Different letters indicated significant (p < 0.05) differences.

### H_2_O_2_ and MDA content

In Arta cultivar, H_2_O_2_ content augmented at 2500 and 5000 µM Ce and then decreased to lower values than that of control at other doses of Ce treatment. H_2_O_2_ content in Baharan cultivar reduced significantly following treatment with Ce at all concentrations as compared to control (Table 3). Application of varying Sm doses to Arta and Baharan cultivar significantly declined H_2_O_2_ content and maximum decrease showed at 15,000 µM (Table 4).

The 2500 µM Ce treatment did not cause any significant impact on MDA content in both cultivars. The MDA content at 5000, 7500, 10,000 and 15,000 µM Ce treatment was 114.28, 265.71, 258.57 and 285.71% in Arta cultivar and 75, 125, 211.25, 200% in Baharan cultivar larger than the control (Table 3). Treatment with 2500 µM Sm had no significant effect on the MDA content in both cultivars. The MDA content showed a significant rise by 35.89, 125.64, 228.20 and 269.23% in Arta cultivar and 75, 90.90, 125, 186.36% in Baharan cultivar at 5000, 7500, 10,000 and 15,000 µM Sm treatment as compared to control (Table 4).

### Enzymatic antioxidant system

**Figure 1:**
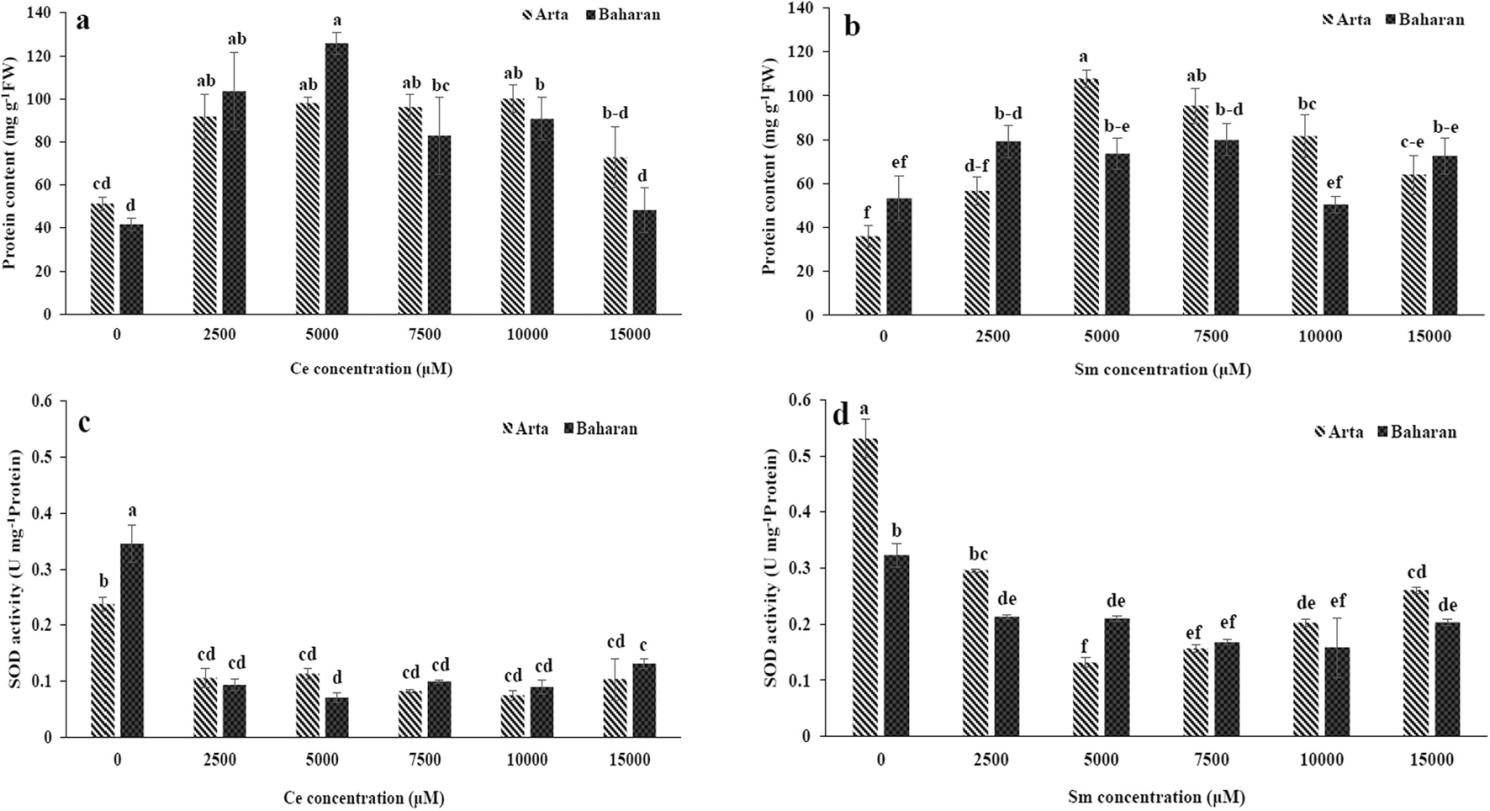
Effect of Ce and Sm on protein content (**a**,**b**) SOD activity (**c**,**d**) in wheat plants. Vertical bars indicate Means ± SE based on three replicates. Different letters above columns indicated significant (P < 0.05) differences.

Ce treatment boosted protein content in both cultivars (Fig. 1a). Sm treatment also caused an increase in the protein content in Arta cultivar, whereas this was not caused significant change trend in Baharan cultivar (Fig. 1b).

**Figure 2:**
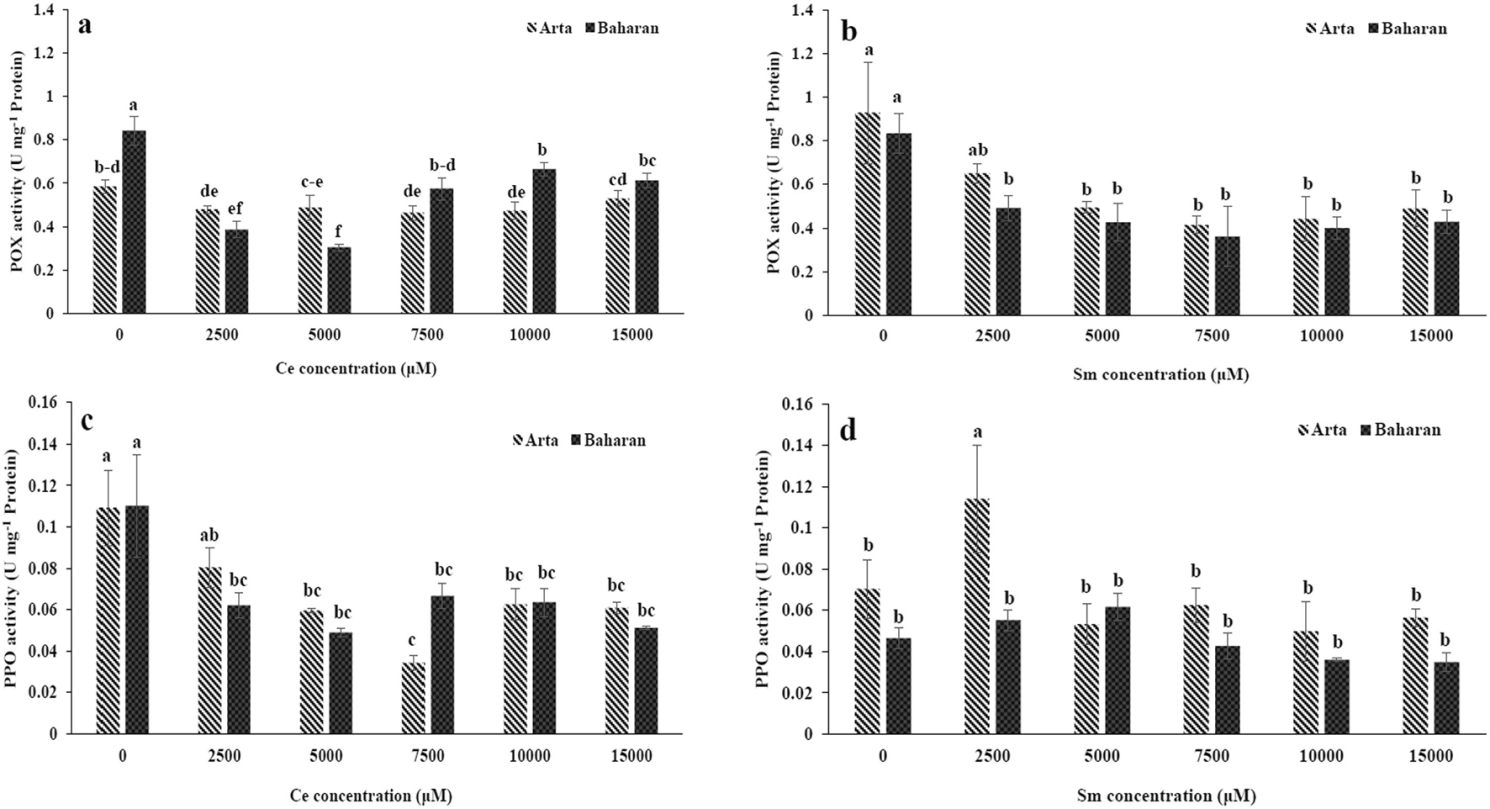
Effect of Ce and Sm on POX activity (**a**,**b**) and PPO activity (**c**,**d**) in wheat plants. Vertical bars indicate Means ± SE based on three replicates. Different letters above columns indicated significant (P < 0.05) differences.

The activities of the ROS scavenging enzymes such as SOD, POX and PPO are shown in Figs. 1, 2. Ce treatment considerably declined SOD activity in both cultivars as compared to that of control and no significant difference was found in the SOD activity between various levels of Ce. Reduction in SOD activity of Baharan Cultivar was more than Arta cultivar (Fig. 1c). No significant difference was found between the POX activity in Ce-treated and control plants in Arta cultivar. A decrease was seen in POX activity of Baharan cultivar under Ce treatment as compared to control (Fig. 2a). PPO activity was significantly reduced in two cultivars at all Ce doses (Fig. 2c).

All of Sm treatments dropped SOD activity in both cultivars, but this reduction was higher in Arta cultivar when compared to Baharan cultivar (Fig. 1d). A decrement in POX activity occurred in all levels of Sm treatment in two cultivars and no significant changes in POX activity were observed between different concentrations (Fig. 2b). PPO activity enhanced in Arta cultivar by 2500 µM Sm and then decreased at higher concentrations. Treatment with all tested Sm concentrations induced no significant variations in PPO activity of Baharan cultivar (Fig. 2d).

### Non-Enzymatic antioxidant system

**Figure 3:**
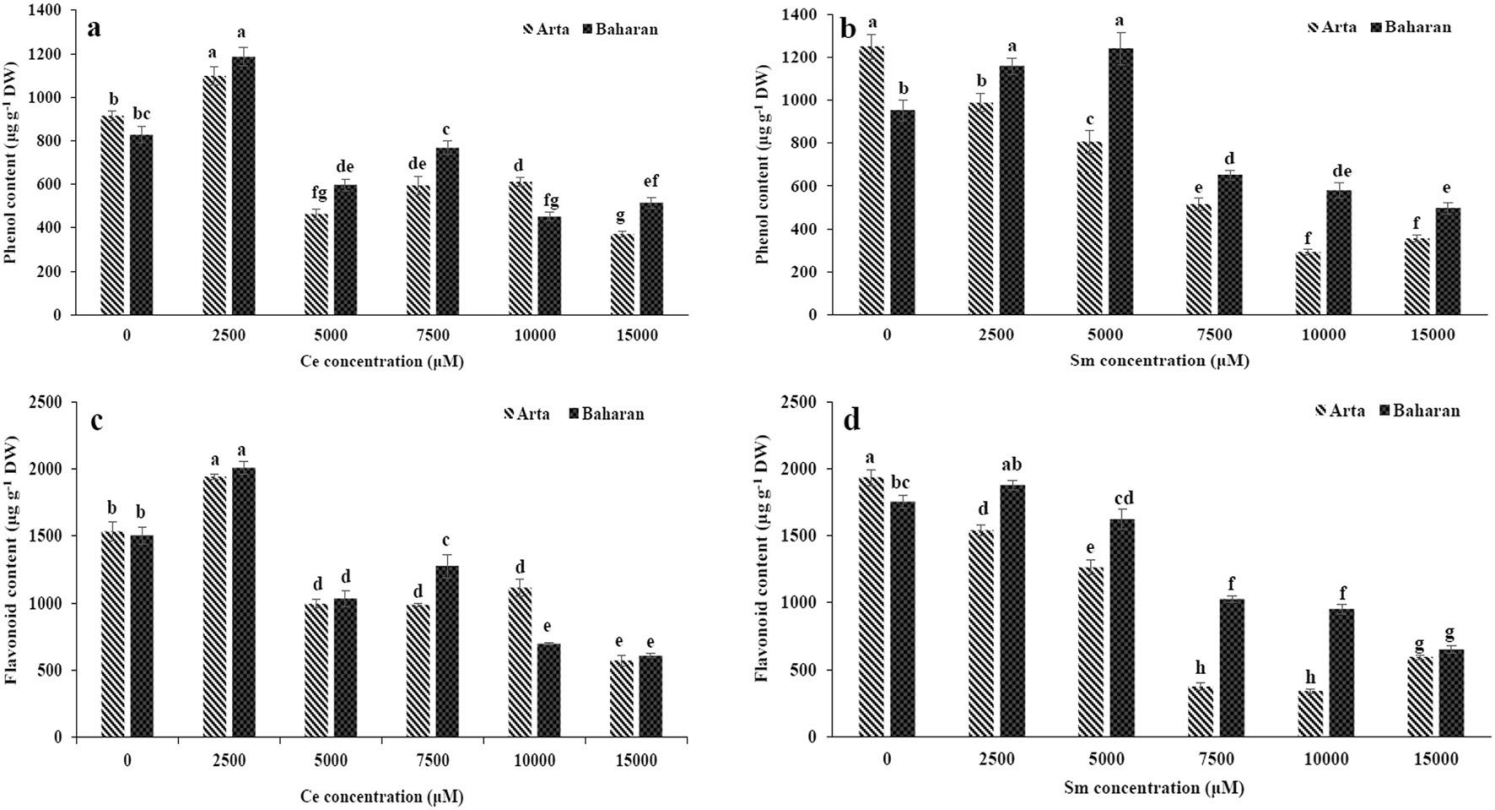
Effect of Ce and Sm on total phenol (**a**,**b**) and flavonoid (**c**,**d**) of wheat plants. Vertical bars indicate Means ± SE based on three replicates. Different letters above columns indicated significant (P < 0.05) differences.

**Figure 4:**
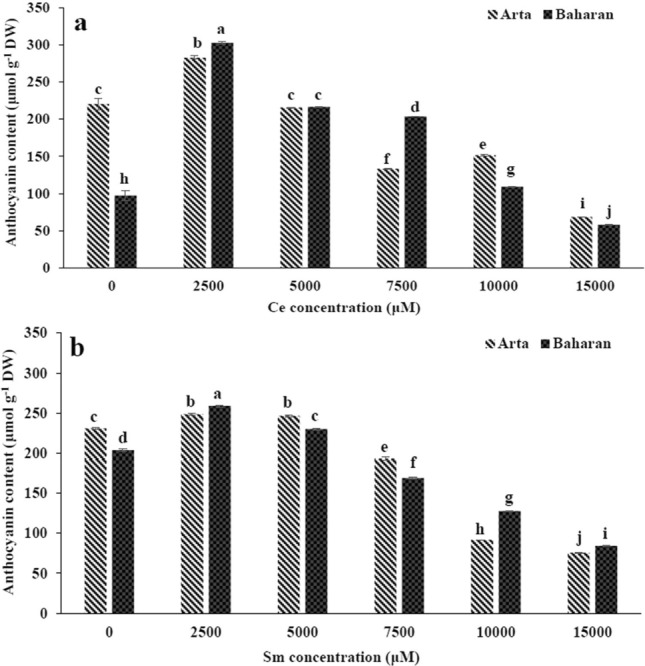
Effect of Ce and Sm on anthocyanin content (**a**,**b**) in wheat plants. Vertical bars indicate Means ± SE based on three replicates. Different letters above columns indicated significant (P < 0.05) differences.

The effect of Ce and Sm on the non-enzymatic antioxidants of two wheat cultivars is displayed in Figs. 3, 4. Total phenol content was heightened under Ce treatment up to 2500 µM in both cultivars; the effect of Ce on total phenol content was more pronounced in Baharan cultivar (Fig. 3a). Flavonoid content in both cultivars boosted in plants treated with 2500 µM Ce, but in the plants treated with other doses of Ce a significant decline was observed (Fig. 3b). Anthocyanin content only enhanced by 2500 µM Ce whereas it reduced in both cultivars at 5000–15,000 µM Ce as compared to non-treated plants (Fig. 4a).

The total phenol content in Arta cultivar was significantly reduced and lower than that of the control under Sm treatments. Total phenol content was higher in Baharan cultivar exposed to 2500 and 5000 µM Sm comparison with the control, whereas higher doses caused significant decrease in this content (Fig. 3b). A significant decrease in flavonoid content in Arta cultivar was detected under Sm treatments. Treatment with 7500–15,000 µM Sm declined flavonoid content in Baharan cultivar, while no significant differences were found at other Sm treatments, compared with the control (Fig. 3d). Similar results were observed for anthocyanin content in both cultivars under Sm treatment. In both cultivars, anthocyanin content increased at 2500 and 5000 µM Sm, then decreased up to 15,000 µM Sm to higher values than that of control (Fig. 4b).

### Investigation of correlation between various Ce and Sm levels and studied parameters by PCA analyze

**Figure 5:**
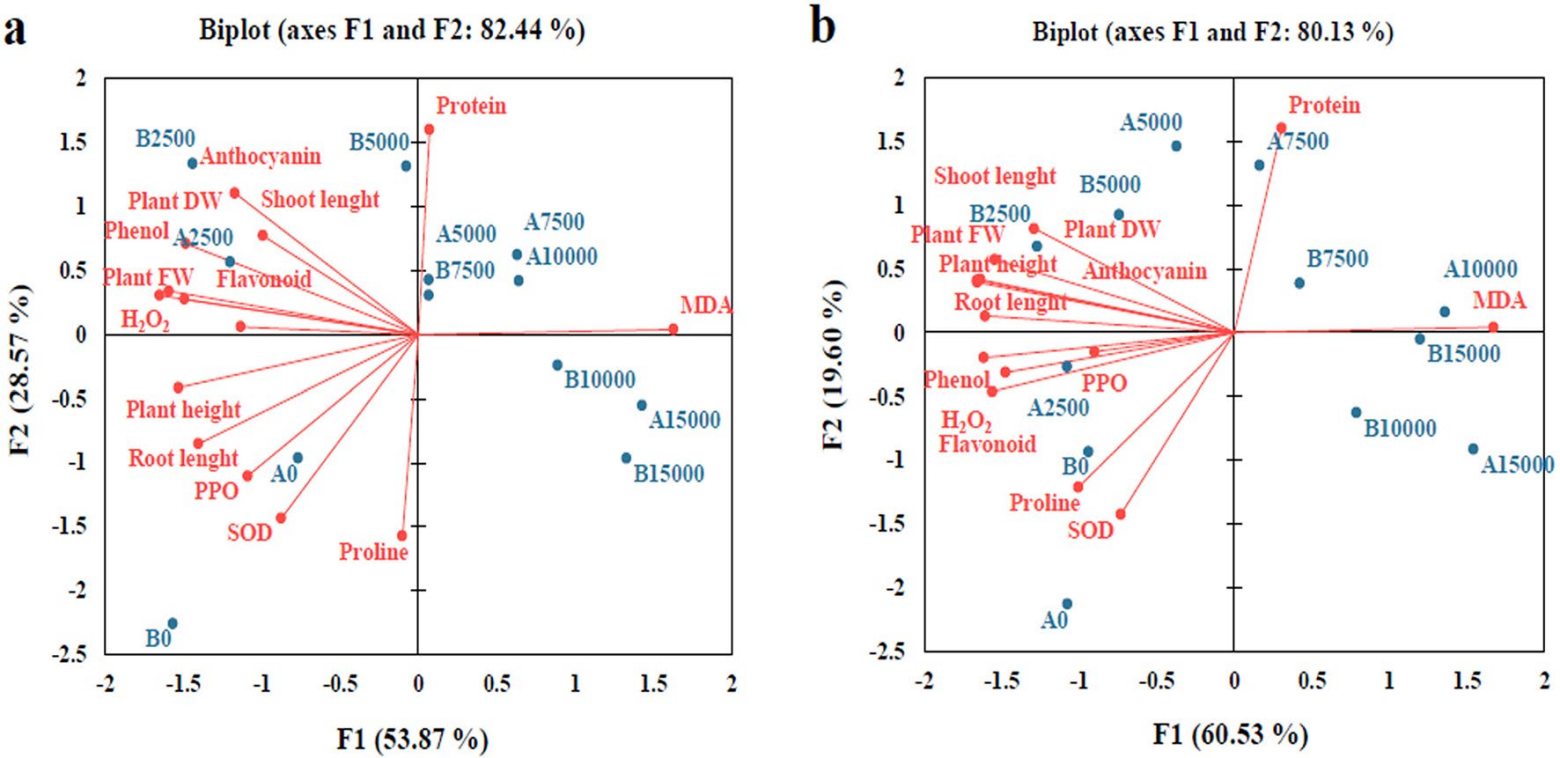
Principal component analysis (PCA) of physiological parameters under Ce (**a**) and Sm (**b**) treatment.

The potential correlations among the studied variables under the different treatments of Ce and Sm were analyzed based on Pearson's correlation coefficient. According to this analyze, DW or growth in wheat plants treated with Ce and Sm had more positive correlation with non-enzymatic antioxidant system than that enzymatic antioxidant system. Thus, these findings confirmed that that non-enzymatic antioxidant mechanism is more important for cope on destructive effects of Ce and Sm in wheat plants. In both treatments, growth negatively correlated with MDA level. The control treatment adjudged as the best value giving treatments. Its impact was followed by that of 2500 µM of Ce and Sm. This displayed the stimulatory effect of 2500 µM. The other doses caused growth limitations in wheat plants as these treatments were clustered on the other side of loading plot (Fig. 5a,b).

## Discussion

Numerous studies have revealed that the elevation of growth and development in plants happens under low levels of REEs, and an inhibitory impact has been witnessed under high levels^15,16^. Our results indicated that the negative or positive impacts of Ce and Sm on growth depended on the dosage of Ce and Sm. We found that two cultivars did not show any signs of damage and that the growth of the plants enhanced when the Ce and Sm concentration was 2500 µM. However, when the Ce and Sm concentration exceeded 2500 µM, the growth of two cultivars was decreased and significantly lowers than those of the control group. These findings indicated that Ce and Sm had a hormesis impact on growth in wheat plants. Based on our results, negative effect of Ce and Sm was more on the root than on the shoot. Also, our findings recommended that two cultivars can show a different sensitivity to Ce and Sm. Arta cultivar was more resistant to Ce, but Baharan cultivar was more resistant to Sm. In our work, appropriate concentrations of Ce (2500 µM) and Sm (2500 µM) effectively enhanced the non-enzymatic antioxidant system of both cultivars and declined the negative effect of Ce and Sm, and subsequently increased growth. However, high concentrations of Ce and Sm blocked enzymatic antioxidant system and induced oxidative damage, and severely depressed the plant growth. Ce affect metabolism in *Lemna minor* L. with a biphasic trend, with stimulatory impacts at lesser levels and inhibitory impacts at upper levels^17^. The stimulated growth in REEs-treated plants is directly correlated to cell division^18^, enhancement of mitotic rate^19^ and augmentation of the absorption of valuable ions for plants^20^. However, study of some molecular parameters and the genes involved in oxidative stress resistance in reaction to Ce and Sm poisonousness, would aid to more understand of adaptation strategies adopted by this plant to decrease Ce and Sm stress.

Proline accumulates in plants in response to water, salinity and heavy-metal stresses. The enhancement of proline content by plants is a defensive strategy. Proline acts such as a source of carbon and nitrogen, ROS scavenger, osmotic buffer, osmoprotectant and membrane preservative^21^. The impacts of Ce and Sm on the proline content of wheat plants follow a dose-depending response with an induction at high concentrations and a reduction at low concentrations. This biphasic impact of Ce and Sm on proline accumulation is consistent with data published for *Vigna unguiculata*^22^. REEs are recommended to be complicated in enhancing the water consumption efficiency in plants by inducing their proline amount. Accumulation of proline in plants treated with Ce may involve to steadying subcellular components and osmotic equilibrium in the cell. The more content of proline detected in plants treated with higher levels of Ce, can be connected with enhance ability of suppressing ROS and defending cell against oxidative injury as a defense strategy^23^.

High doses of REEs may lead the ROS production and cause to oxidative stress^24^ in plants. Antioxidant enzymes such as SOD, PPO and POX play a main function in the protective responses of plants. SOD activation is first line of protection against ROS under stresses, which changes superoxide anion to H_2_O_2_^25^, while POX enzyme converts H_2_O_2_ to O_2_ and H_2_O. The specific enzymes do not react similarly to all metals. Several elements cause to an enhance in enzymes activities, whereas other elements decline the enzymes activities^26^. The excess ROS can cause lipid peroxidation (assayed as MDA content) by demeaning polyunsaturated fatty acids. The observed changes of lipid peroxidation and antioxidant system are typical indicators of REEs toxicity^27^. In our experiment, Ce and Sm treatments reduced enzymatic antioxidant system (SOD, POX and PPO) were concomitant to increased levels in lipid peroxidation (MDA content) in both cultivars, which can be regarded as typical indicator of cellular injury. Therefore, lipid peroxidation as well as disturb of antioxidant systems, i.e. usual indicators for stress conditions, happened after treatment with Ce and Sm in wheat plants. The increase in MDA level was associated with reduction in growth, thus confirming the potential danger in wheat plants. Our data proposed that the scavenging roles of POX and SOD were impaired. Chen et al.^11^ suggested that the declined activity of SOD at upper Ce concentrations was incompetent to remove superoxide anion leading to boosted membrane hurt. Also, the higher dose of Ce treatment, the less effective enzymes were at suppressing ROS and the more injury to the cells.

Environmental stresses, comprising metallic stress, could augment production non-enzymatic antioxidants in plants to decline the generation of ROS and escape cell injury. Low doses of Ce and Sm induced non-enzymatic antioxidants included phenol, flavonoid and anthocyanin content, while high doses of these elements reduced accumulation of metabolites. Thus, the accumulation of non-enzymatic antioxidants was significantly dependent on the dose of Ce in wheat plants in this work. These observed alterations in non-enzymatic antioxidants might be a self-protection strategy to mitigate the metallic stress in wheat plants. Many investigators have illuminated the antioxidant ability of flavonoids and phenols in chelating uptake elements and suppressing ROS by transmitting electrons to free radicals. Metals have the capacity to decline lipid hydroperoxide (LOOH) by the hemolytic breakdown of the (O–O) bond, subsequent in lipid alkoxyl radicals and the formation of free-radical chain oxidation. Flavonoids and phenolic compounds can trap lipid alkoxyl radicals to prevent lipid peroxidation^28^. Our data are in agreement with the work of Dridi et al.^22^, where Ce-treated *Helianthus Annuus* plants showed a remarkable accumulation of flavonoid and phenolic compound. PAL is a main enzyme which controls phenylalanine biosynthesis into phenolic compounds^30^. Chen et al.^11^ showed that the maximum PAL activity was detected at moderate concentrations of Ce (0.5–1.0 mM) and was agreement with the enhancement of flavonoid content in Ginkgo suspension cells. Thus, REEs can increase phenolic compounds by increasing PAL activity. Also, REEs enhance the generation of secondary metabolites by promoting the transcriptions of critical biosynthetic genes^31^.

## Conclusion

Ce and Sm influenced growth in wheat plants following a biphasic trend**,** with stimulatory impacts at lower doses and inhibitory impacts at higher doses. High doses of Ce and Sm induced proline accumulation in wheat plants. Ce and Sm treatments blocked antioxidant enzymes and enhanced lipid peroxidation. Change in non-enzymatic antioxidant compounds was detected in wheat plants under Ce and Sm exposure, with a concentration-dependent trend. Although the complete mechanism by which Ce and Sm are taken up by plants residues to be studied, we have presented some novel insights into the physiochemical mechanisms of plants in a Ce-enriched environment or Sm-enriched environment beyond mere accumulation investigates. Furthermore, future investigations will be required to recognize the molecular processes responsible for the biochemical and physiological reactions perceived.

## Materials and Methods

### Plant material and experimental plan

All the plant experiments were in compliance with relevant institutional, national, and international guidelines and legislation. Seeds of two cultivars of wheat (Arta and Baharan) were obtained from the Seed and Plant Improvement Research Institute, Karaj, Iran, and used for the experiments. Seeds of two cultivars were sown in plastic pots containing perlite. After germination, seedlings were irrigated with equal amount of half strength Hoagland solution (pH = 6.8) for 6 days. Seedlings were grown in greenhouse with a 16 h light/8 h dark cycle at 25/18 °C day/night temperatures. Then, the pots were then divided into twelve groups of three pots each. The pots were irrigated with 100 mL half strength Hoagland solution at alternative days. To test the effects of different Ce and Sm concentrations, pots were treated with various concentrations of Ce(NO_3_)_3_.6H_2_O (0, 2500, 5000, 7500, 10,000 and 15,000 μM) and Sm(NO_3_)_3_.6H_2_O (0, 2500, 5000, 7500, 10,000 and 15,000 μM) at alternative days for one week. Then pots were irrigated with half strength Hoagland solution for two weeks and plants were collected for analyses in all the experiments.

### Growth parameter and proline content

The plants were evaluated one week after being exposed to Cerium and Samarium in terms of fresh weight (FW), dry weight (DW) and other parameters. For the proline content assessment according to Bates et al.^32^, 0.1 g of leaf tissue was homogenized in 3 mL sulfosalicylic acid (3%) and then was centrifuged at 13,249×*g* for 20 min. Then, 0.5 mL of supernatant was combined with acid ninhydrin (1 mL) and glacial acetic acid (1 mL) and then was boiled at 100 °C for 1 h. The reaction mixture was extracted with 2 mL toluene and the absorbance was assayed at 520 nm via l-proline as a standard.

### Measurement of H_2_O_2_ level

The hydrogen peroxide (H_2_O_2_) level was estimated by Velikova et al.^33^ manner. Leaf tissue (0.5 g) was homogenized in 0.1% TCA then was centrifuged at 7840 × g for 10 min. Supernatant (0.5 mL) was mixed to 0.5 mL potassium phosphate buffer (pH 7.0) and 1 mL potassium iodide (1 M) and absorbance was assayed at 390 nm. Standard curve was prepared with different levels of H_2_O_2_.

### Lipid peroxidation

To assay the lipid peroxidation, a testosterone (TBA) test, which identifies MDA as the final product of lipid peroxidation was conducted according to Heath and Packer^33^. Leaf tissue (0.5 g) was homogenized in 0.1% TCA and then was centrifuged at 7840×*g* for 10 min. The supernatant (0.5 mL) was added to 1 mL of thiobarbituric acid (0.5%) in 20% TCA. The mixture was heated in 95 °C for 30 min and then was centrifuged at 7840×*g* for 10 min. The absorbance of supernatant was assayed at 532 and 600 nm.

### Enzymatic antioxidants and protein content

Leaf material (0.5 g) was extracted at 4 °C with 1 M Tris–HCl (pH 6.8) to estimate different enzyme activities. The homogenate was centrifuged at 13,249×*g* for 20 min at 4 °C and the obtained supernatant was kept at − 70 °C and later used for enzyme assays. Protein content was estimated based on Bradford^35^ method.

The superoxide dismutase (SOD) activity was measured according to Giannopolitis and Ries^36^ method. Reaction solution comprised potassium phosphate buffer (50 mM), 0.1 mM EDTA, 75 μM NBT, 13 mM methionine, 75 μM riboflavin and 100 μL of enzyme extract. The reaction solution was placed in front of the light for 18 min and absorbance was assayed at 560 nm.

For assessment of peroxidase (POX) activity, reaction mixture comprised 0.2 mL H_2_O_2_ (3%), 0.1 mL benzidine (40 mM), 2 mL of 0.2 M acetate buffer (pH 4.8) and 50 μL of enzyme extract. Activity of this enzyme was measured at 530 nm^37^.

The activity of polyphenol oxidase (PPO) was determined according to Raymond et al.^38^. The reaction mixture included 2.5 mL of 200 mM potassium phosphate buffer (pH 6.8), 0.2 mL of 20 mM pyrogallol and 20 μL enzyme extract. The enzyme activity was assayed at 430 nm.

### Non-enzymatic antioxidants

In order to preparation of methanolic extract, 0.1 g of dry tissue was homogenized in 5 mL methanol 80% and then was centrifuged at 5000×*g* for 20 min. For the total phenol content measurement, 0.1 mL methanolic extract was added with 2.5 mL Folin–Ciocalteu reagent 10%. The mixtures were neutralized by sodium bicarbonate 7% and then absorbance was measured at 765 nm^39^.

Content of flavonoid was determination by Chang et al.^40^ method. In this method 0.1 g of leafs was homogenized in 2 mL of methanol 80%. Methanolic extract (0.5 mL) was mixed with 1.5 mL of methanol 80%, 0.1 mL of aluminium chloride (10%), 0.1 mL of potassium acetate (1 M) and 2.8 mL of distilled water and the absorbance was assayed at 415 nm after 30 min.

The content of anthocyanin was measured in 0.3% HCl in methanol at 25 °C using the extinction coefficient (33 cm^2^ mol^-1^) at 550 nm^41^.

### Statistical analyses

Each experiment was repeated three times and the data were analyzed by either one- or two-way analysis of variance (ANOVA) using SPSS (ver. 26). Means were compared by Duncan’s test at the 0.05 level of confidence. Principal component analysis (PCA) analysis was used for obtaining correlation matrix, giving the Pearson’s correlation coefficients between each pair of variables, i.e. the analytical parameters tested. PCA analysis was done by XLSTAT (2016).

## Data Availability

All data generated or analyzed during this study are included in this published article**.**
